# Myxofibrosarcoma metastasis to the pterygopalatine fossa: A case report

**DOI:** 10.1016/j.amsu.2020.10.025

**Published:** 2020-10-20

**Authors:** Haya Deeb, Afaf Ahmad, Areej AlAssaf

**Affiliations:** a Faculty of Medicine, Damascus University, Syria; b Al-Mouassat University Hospital, Syria

**Keywords:** Myxofibrosarcoma, Fibrosarcoma, Pterygopalatine fossa, Partial maxillectomy, Metastasis, MFS, Myxofibrosarcoma, MFH, Malignant fibrous histocytoma, MRI, magnetic resonance imaging, CT, Computed tomography

## Abstract

**Introduction:**

Myxofibrosarcoma (MFS) is a rare subtype of a malignant soft tissue tumor that occurs mainly in adults, and peaks at the age of 70. It typically presents as a slow growing, painless mass in the proximal part of the extremities. It is characterized with a high recurrence rate and a low rate of distant metastases; the most common metastases site is the lungs, and in some extremely rare cases it was mentioned that there was metastases to the head and neck region. We here report the first case of a myxofibrosarcoma metastasized from the gluteal region to the pterygopalatine fossa, which is the first report in the literature of this rare metastatic spread of myxofibrosarcoma.

**Case presentation:**

a 70 year-old male presented with diplopia and limited right eye movement. His medical history was significant for myxofibrosarcoma in his gluteus maximus. Magnetic resonance imaging showed a low signal mass in the pterygopalatine fossa. The tumor neither invaded the maxillary bone nor the maxillary sinus; the therapy plan was resection of the mass by partial maxillectomy followed by adjuvant radiotherapy.

**Conclusion:**

Metastasis to the pterygopalatine fossa should be considered in a patient with myxofibrosarcoma history presents with neuro-opthalmic symptoms. Partial maxillectomy in tumors that do not infiltrate into adjacent structures should be considered as a minimally invasive therapy.

## Highlights

• Myxofibrosarcoma can rarely metastasize to the pterygopalatine fossa from the lower limb.
• Developing diplopia after lower limb sarcoma may refer to metastasis to the head.
• Less invasive interventions are recommended when no bone involvement is present.
• Good patient consult and psychological support is essential for optimal outcomes.

## 1 Introduction

The term “Myxofibrosarcoma” (MFS) refers to a connective soft tissue neoplasm of fibroblastic origin. It was first described in 1977 by Angervall et al. [1,2], and was classified as a myxoid variant of malignant fibrous histiocytoma (MFH). MFS is the second most common subtype of MFH [3]. This tumor is commonly found in proximal parts of extremities in elderly adults [4], which its peak in the seventh decade of life [5]. It classically manifests as a slow-growing, painless mass in extremities, with high local recurrence rate of 27% and low distant metastatic potential of 23% [6]. Metastasis is predominantly hematologic, with the lungs being the most common site [7]. Its local reoccurrences depend on a variety of factors such as the tumor's grade, achieving surgical negative margins and administration of adjuvant radiotherapy [8]. According to the previously conducted studies, MFS has a rare occurrence in the head and neck areas, with only 19 reported cases of primary tumors located in the maxillary sinus, the sphenoid sinus, and the parotid gland [5]. MFS diagnosis is challenging, especially when encountered in uncommon locations [9] like the pterygopalatine fossa. Pterygopalatine fossa neoplasms are either primary tumors originating in the fossa or extend to the area by direct invasion or via neurovascular routes. The most common tumor type that occurs in the fossa is squamous cell carcinoma, with the mean age of diagnosis being 60 years old [10].

In this report, we are presenting a rare metastatic spread of MFS from the left gluteus to the pterygopalatine fossa. To the best of our knowledge, this is the first article that reports this rare localization of the MFS metastasis. MFS's clinical presentation, histological features, and its surgical treatment are also discussed. This work was done in compliance with SCARE checklist [11].

### 1.1 Case presentation

In September 2019, a 70 year-old male patient presented with diplopia in his right eye that progressed during the last two months, in addition to limited right eye movement and right temporal headache. His past medical history included the surgical removal of myxofibrosarcoma tumor from his left gluteal region 5 years ago. It was treated by neo-adjuvant radiotherapy followed by wide local surgical excision and finally by adjuvant radiotherapy. The ophthalmic examination revealed a diplopia and paralysis of abducens nerve in his right eye, normal left eye examination, normal visual acuity and normal appearance of optic disc of both eyes.

**Fig. 1:**
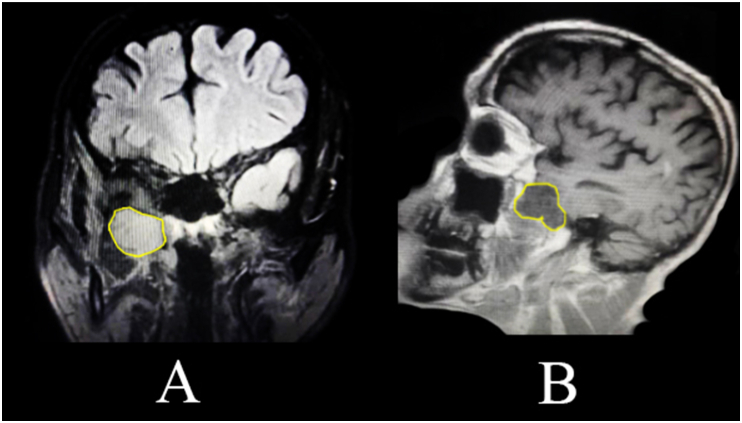
**(A)** The yellow circle shows a low signal mass in T1-Weighted MRI image with contrast. (**B)**The yellow circle shows a high signal mass in T2-Weighted MRI. (For interpretation of the references to colour in this figure legend, the reader is referred to the Web version of this article.)

Head and neck MRI revealed a low signal mass in T1-Weighted image with contrast (Fig. 1-A) and High signal in T2-Weighted image (Fig. 1-B), measuring 4 × 5x2 cm in his right pterygopalatine fossa and extending superiorly to the right orbital fissure. This extension of the tumor compressed the abducens nerve, causing diplopia. A CT scan with contrast was performed to screen head, neck and chest but no other tumors or metastases were detected. Based on these findings, the decision was made to remove the tumor surgically.

**Fig. 2:**
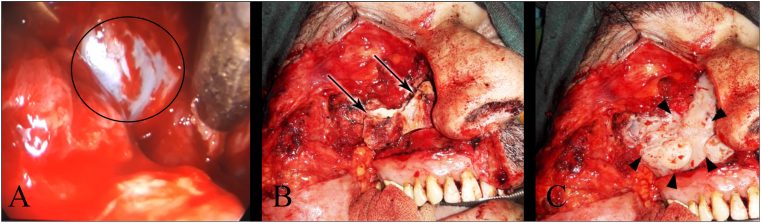
**(A)** Endoscope image shows the round foramen and meninges were not invasive by the tumor (the black circle) **(B)** The returned bone flap with the wires that went through pre-made holes (arrows) **(C)** The bone wax was used to fill the bone's sutures (pointers).

Under general anesthesia, a skin incision was made on the anterior margin of the sternocleidomastoid muscle at the level of the hyoid bone to expose the right external carotid artery. It was ligated superiorly to the branching point of the superior thyroid artery to avoid excessive bleeding. A Weber-Fergusson incision was made from the middle of the upper lip, along the nasofacial groove to the medial canthus, and the incision was extended below the subcilliary line to the lateral canthus. The skin and musculature layers in the area were swung laterally to expose and ligate the infra-orbital neurovascular bundles. The bone flap of the anterior surface of the maxillary sinus was removed separately. The posterior and medial walls of the maxillary sinus along with the perpendicular plate of the palatine bone were swung laterally, and the internal maxillary artery was ligated and cauterized. The tumor in the pterygopalatine fossa was completely isolated and the maxillary nerve (V2) was intact. The tumor was resected with the posterior wall of the maxillary sinus using the standard partial maxillectomy approach. The dissection was carried on until the round foramen and dura mater were reached. An endoscope was used to make sure the entire tumor was resected (Fig. 2-A). The removed bone flap was returned and fixed in its place using wires that went through pre-made holes (Fig. 2-B) and the bone's sutures were filled with bone wax (Fig. 2-C).

**Fig. 3:**
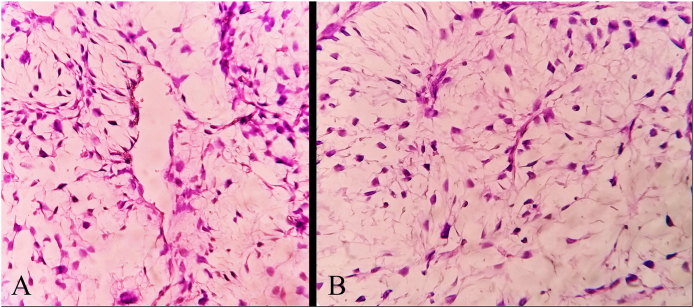
**(A)** The tumor under the microscope showed the considerable curvilinear reactive vascularity in a myxoid stroma. **(B)** The microscopic examination revealed moderate pleomorphic spindle cells in variable shapes and sizes in a myxoid stroma with nuclear atypia, scattered mitosis.

**Fig. 4:**
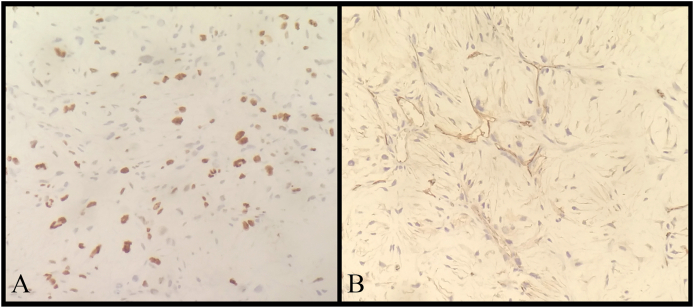
(A) Ki-67 proliferative index marker of the tumor was >20%. (**B)** The tumor was immune positive for CD34.

Gross examination showed a white soft tissue mass. The microscopic examination revealed moderate pleomorphic spindle cells in variable shapes and sizes in a myxoid stroma with nuclear atypia, scattered mitosis, and a considerable curvilinear reactive vascularity. These findings were compatible with a grade 2 myxofibrosarcoma (Fig. 3 – A,B). Immunohistochemically, the tumor was immunopositive for CD34 (Fig. 4-B). Whereas, it was immunonegative for S100. Ki-67 proliferative index marker was >20% (Fig. 4-A). The pathology lab was unable to detect the microscopic negative margins because the tumor was resected gradually and in pieces.

The patient was scheduled for having 30 sessions of adjuvant radiotherapy 5 days a week for 6 weeks. Unfortunately, the patient did not start the treatment on time and did not fully commit to it. So after four months of the surgery, the tumor recurred at the same location of the previous surgery. However, the patient refused to receive any additional treatment. After 6 months of follow up, his general health status was still stable.

## 2 Discussion

Myxofibrosarcoma presenting in the head and neck region is extremely rare. We can conclude that the tumor of this case had metastasized to this area because of the patient's history of having a myxofibrosarcoma 5 years ago, putting him in the window range of having recurrent distant metastasis. According to a recent study, the range of having distant metastatic recurrence of the MFS is 2.5 months–7.7 years [6].

The unique symptomatology that the patient presented with in this case is attributed to the rare localization of the tumor in the pterygopalatine fossa. Whereas in the majority of cases that presented with diplopia manifested as a mass in the mid-cheek or temporomandibular joint areas [12].

MFS has a low potential of giving distant metastasis. When it metastasizes, it is usually via the hematologic route rather than the lymphatic one. The lungs are the most common destination of MFS metastases [7]. Malignant tumors that metastasize to the pterygopalatine fossa are very rare. Some previous studies reported metastatic tumors to the pterygopalatine fossa: the first was a metastatic neuroendocrine breast cancer and it was only treated with radiation therapy and the second was a metastatic renal cell carcinoma, which was treated with both radiotherapy and chemotherapy [13,14]. MFS metastasis to the pterygopalatine fossa has not been mentioned before, making this case the first with this rare metastasis site.

The complete resection was very challenging due to anatomical complexity of pterygopalatine fossa. Total maxillectomy, combined with anterior craniotomy have been used as a standard procedure for such cases, resulting in significant postoperative masticatory and cosmetic problems [12]. On the other hand, metastatic tumors to pterygopalatine fossa were treated with radiotherapy only [13]. In this case, the tumor neither invaded the maxillary bone nor the maxillary sinus, allowing for the performance of partial maxillectomy with macroscopic negative margins to resect the tumor. This procedure maintains the normal function of facial muscles and cosmesis resulting in better quality of life. Partial maxillectomy is the procedure of choice for tumors in this area. Pathology lab results and immunohistological stains distinguish MFS among other possible differential diagnosis: Malignant fibrous histiocytoma, Myxoid liposarcoma, and Myxoid dermatofibrosarcoma.

Distant metastasis of MFS occurs in 20% of the cases with a third grade tumor and 4% in patients with a second grade tumor [15]. We highly suspect that the lack of patient's commitment to the scheduled radiotherapy sessions attributed to the recurrence of the tumor at the site of the metastasis, because radiotherapy is highly important when partial maxillectomy is performed.

## 3 Conclusion

In conclusion, we reported this case to highlight that metastasis to the pterygopalatine fossa should be considered when a patient with myxofibrosarcoma history presents with neuro-opthalmic symptoms and signs.

We also recommend partial maxillectomy as a minimally invasive therapy with adjuvant radiotherapy, to resect tumors in the pterygopalatine fossa that do not aggressively invade the maxillary bone and sinus. This procedure preserves the visceral cranial bones and the associated neurovascular bundles.

## Ethical approval

No ethical approval was needed.

## Patient's consent

Written patient consent was obtained before reporting this case.

## Availability of data and materials

All data are available from the corresponding author on reasonable request.

## Provenance and peer review

Not commissioned, externally peer reviewed.

## Declaration of competing interest

All the authors declared that they have no conflicts of interest.
